# Body Composition of Bangladeshi Children: Comparison and Development of Leg-to-Leg Bioelectrical Impedance Equation

**DOI:** 10.3329/jhpn.v30i3.12291

**Published:** 2012-09

**Authors:** I. Khan, Ashraful Hawkesworth, Sophie Mohammad Delwer Hossain Hawlader, Shams El Arifeen, Sophie Moore, Andrew P. Hills, Jonathan C. Wells, Lars-Åke Persson, Iqbal Kabir

**Affiliations:** ^1^icddr,b, GPO Box 128, Dhaka 1000, Bangladesh; ^2^International Maternal and Child Health, Department of Women's and Children's Health, Uppsala University, Sweden; ^3^Medical Research Council International Nutrition Group, London School of Hygiene & Tropical Medicine, London, United Kingdom; ^4^Institute of Health and Biomedical Innovation, Queensland University of Technology, Brisbane, Queensland, Australia; ^5^Childhood Nutrition Research Centre, 30 Guilford Street, London WC1N 1EH, UK

**Keywords:** Bioelectrical impedance, Body composition, Children, Deuterium oxide dilution, Bangladesh

## Abstract

The aim of this study was to investigate the validity of the Tanita TBF 300A leg-to-leg bioimpedance analyzer for estimating fat-free mass (FFM) in Bangladeshi children aged 4-10 years and to develop novel prediction equations for use in this population, using deuterium dilution as the reference method. Two hundred Bangladeshi children were enrolled. The isotope dilution technique with deuterium oxide was used for estimation of total body water (TBW). FFM estimated by Tanita was compared with results of deuterium oxide dilution technique. Novel prediction equations were created for estimating FFM, using linear regression models, fitting child's height and impedance as predictors. There was a significant difference in FFM and percentage of body fat (BF%) between methods (p<0.01), Tanita underestimating TBW in boys (p=0.001) and underestimating BF% in girls (p<0.001). A basic linear regression model with height and impedance explained 83% of the variance in FFM estimated by deuterium oxide dilution technique. The best-fit equation to predict FFM from linear regression modelling was achieved by adding weight, sex, and age to the basic model, bringing the adjusted R^2^ to 89% (standard error=0.90, p<0.001). These data suggest Tanita analyzer may be a valid field-assessment technique in Bangladeshi children when using population-specific prediction equations, such as the ones developed here.

## INTRODUCTION

Body composition measurement in children is important for assessing nutritional status for both health and disease (1). Determination of body fatness by measuring body composition in younger age-groups has been shown to be an important risk factor for later disease (2,3). A study undertaken by Yajnik (4) in India compared full-term babies born in India with those born in the UK and found that Indian babies were relatively lighter, shorter, and thinner. The thinness of the Indian babies is related to a paucity of lean soft tissues, including abdominal viscera and skeletal muscle. The characteristic South Asian body phenotype, which comprises low muscle mass, high percentage of body fat, and tendency to central adiposity, is different from that in other populations (5,6). A better characterization of body proportions and composition during early life may be of relevance for improved understanding of the early origins of diseases in adulthood, and hence, the need for accurate assessment of body composition in children. Various established methods are used for body composition assessment, including air-displacement plethysmography (ADP), dual-energy x-ray absorptiometry (DXA), underwater weighing (densitometry), and magnetic resonance imaging (MRI) (7,8). However, these methods are expensive, not easily portable, time-consuming, and require highly-trained operators, which renders them unsuitable for most field settings.

In epidemiological and field studies, predictions of body fatness are often made from anthropometric measurements, including body mass index (BMI), waist-circumference, waist-hip ratio, and skinfold thickness. However, these techniques do not precisely characterize body fat or muscle mass, and there is a variation across age, sex, and ethnic groups (9,10). Further disadvantages of these techniques include a high degree of both intra- and inter-observer variation and acceptability of the measures in different populations (11).

Bioelectrical impedance analysis (BIA) is a popular and widely-used method of measuring body composition (12). BIA actually determines the electrical impedance of body tissues, which provides an estimate of total body water (TBW) that is converted to an estimate of fat-free mass (FFM), with assumed constant values for the hydration of lean tissue (13,14). BIA is a portable, non-invasive, rapid and relatively inexpensive method for assessing body composition, which lends itself to applications in epidemiological studies. Various BIA machines are available, including the leg-to-leg Tanita TBF-300A analyzer (Tanita Corporation, Tokyo, Japan) (15,16), which measures lower-body impedance as the individual stands on a bathroom-like scale. Although this leg-to-leg BIA differs from traditional arm-to-leg BIA devices, evidence suggests it provides similar body composition measurements to those by the arm-to-leg method (17). The in-built prediction equations used by the Tanita TBF 300A are mainly developed for Caucasian populations, and their validity is questioned when applied to other ethnic groups (18). Different BIA models have been used extensively among various age-groups, and several prediction equations have been developed, though not for the TBF-300A (18-20) and very few for South Asian populations (21).

Thus, there is a need for a valid equation for measuring FFM in Bangladeshi children. Deuterium oxide dilution technique is a safe, non-invasive method that can be used in all population groups, including pregnant women and children (22,23). Therefore, the aim of this study was to assess the accuracy of the Tanita TBF 300A leg-to-leg bioimpedance analyzer in Bangladeshi children and, if necessary, to develop a novel prediction equation for estimation of fat-free mass (FFM), using the deuterium dilution technique as the reference method.

## MATERIALS AND METHODS

### Study site

The study was conducted in Matlab, a poor rural subdistrict located 53 km southeast of Dhaka, the capital of Bangladesh. The International Centre for Diarrhoeal Disease Research, Bangladesh (icddr,b) runs a health and demographic surveillance system (HDSS) that covers a population of about 220,000 and provides health services in the area.

### Study participants

The current investigation was linked to a prenatal nutrition intervention (MINIMat trial—Maternal and Infant Nutrition Interventions in Matlab) with follow-up of the offspring. Full details of the MINIMat trial are published elsewhere (24). For this substudy, 200 children (102 boys and 98 girls) aged 4-10 years were enrolled. Study subjects included a convenient subsample of children enrolled in the MINIMat study, who were 4-5 years old and their older siblings up to 10 years of age. Written informed consent was obtained from the parents/guardians of each participating child. Ethical permission was granted by the Research Review and Ethical Review Committees of icddr,b.

### Data collection

The study was performed at two health subcentres run by icddr,b in the Matlab area. Early in the morning of the day of measurement, a field research assistant accompanied the participants who had fasted overnight to the study locations. Two teams (one team at each subcentre), comprising a medical doctor, a nurse, a field research assistant and a laboratory technician, conducted all measurements. Each measurement was conducted at around the same time of the day. The study was conducted between April and August 2008.

### Anthropometry

Body-weight was recorded to the nearest 0.1 kg with a digital scale (Tanita HD–318, Tanita Corporation, Japan), the participant being in light clothing and bare feet. The scale was calibrated on each study day with a standard 20 kg weight. Height was measured to the nearest 0.1 cm with a daily-calibrated freestanding stadiometer (Leicester Height Measure, Seca 214, UK). Body mass index (BMI) was calculated as weight (kg)/height (m)^2^. Mid-upper arm-circumference (MUAC) was measured to the nearest 0.1 cm with a non-elastic metric measuring tape at the midpoint of the upper arm and with the arm hanging straight at the side of the body. Skinfold thickness was measured in triplicate to the nearest 0.2 mm at four sites (biceps, triceps, subscapular, and suprailiac) with Holtain calipers (Holtain, Crymych, UK): an average of the three skinfold measurements at each site was used. All measurements of skinfold thickness and MUAC were performed in the same order on the left side of the body. Anthropometric measurements (height, weight, MUAC) were done by field research assistant, and skinfold and body composition measurements were performed by the study nurse.

### Bioelectrical impedance

Body composition was assessed by leg-to-leg bioelectrical impedance analysis (12) with a Tanita TBF-300MA Body Composition Analyzer (Tanita Corporation, Tokyo, Japan). BIA measurements were done according to the manufacturer's guidelines at a frequency of 50 kHz. Participants were asked to void their bladder prior to measurement. Height, sex, and age were entered manually; weight was recorded automatically with 0.5 kg as an adjustment for weight of clothes. The Tanita software uses in-built prediction equations to estimate fat mass (FM) and fat-free mass (FFM). These prediction equations are based on Caucasian populations aged 7 years and older. Therefore, the impedance was measured for the whole population while TBW, FM, and FFM from the BIA machine were only used for the age interval 7-10 years.

### Measurement of total body water (TBW) with deuterium dilution

The isotope dilution technique with deuterium oxide (D2O) was used for estimating total body-water (TBW) as described by Colley (25). In brief, this method measures dilution of a known orallyingested dose of the isotope deuterium (D2O) in the body system. Participants orally consumed a dose equal to 0.05 g D2O/kg body-weight. Pre-dose deuterium abundance was obtained from one fasted saliva sample collected on the study day. Saliva (approximately 1 mL) was collected from the children through chewing on a ball of cotton wool, which was then squeezed into a syringe to extract the saliva. The children were instructed to refrain from any food or fluid for at least 30 min before the post-dose saliva samples, which were collected at 3 and 4 h after the administration of deuterium. During the equilibration period, children remained in a specified location with the study team. All saliva samples were stored at −20 ^0^C until shipment to Queensland University of Technology, Australia, for analysis. The enrichment of D2O in the pre-dose and 3, and 4 h post-dose samples was assessed by isotope ratio mass spectrometry, and TBW was subsequently calculated using the mean of 3 and 4 h samples. Deuterium in body water enters other pools within the body, which is known as non-aqueous exchange. The constant 1.041 was used in correcting for non-aqueous hydrogen exchange. The hydration fraction of FFM was assumed dependent on the age and sex of the child as described by Lohman (13,26) and ranged between 76.2% and 78.3%. FFM was calculated from TBW, assuming that FFM has a hydration constant from 76.2% to 78.3% and fat mass was estimated from the difference between body mass and FFM. Weight measured in the early morning and in a fasted state was used for all calculations.

### Statistical analyses

Most statistical analyses were performed with SPSS (version 14.0; SPSS Inc., Chicago, IL, USA), and Analyse-it (version 2.22) free software was used for the Bland-Altman plot analyses. We examined histograms of the dependent variables to confirm that the distributions were Gaussian, and also normality of the fitted variables was assessed using the Shapiro-Wilk test. We did the scatter plot to see the linearity and homoscedasticity or similarity in variance prior to the development of the prediction equations. An all-possible subsets regression analysis was performed for FFM, with the possible independent variables of age, sex, weight, height, BMI, MUAC, impedance, and impedance index included in each analysis. This procedure evaluates the preliminary equations that contain all the possible combinations of independent variables (27). The preliminary equations were selected by measures of goodness-of-fit statistics, including the R^2^ values adjusted for the df and Mallows’ C*p* statistic (28). Mallows’ C*p* statistic is an index of the appropriate number of independent variables in an equation. Ideally, one selects a prediction equation (from a set of possible prediction equations) with the C*p* value that is close to the number of independent variables. A variance inflation factor for each independent variable was also calculated to evaluate multicollinearity (27).

Descriptive statistics were stratified by sex and age, and values expressed as means and standard deviations (SD). Differences between sexes were assessed by independent *t*-tests. Differences across age categories were tested for males and females separately by one-way analysis of variance. For each sex, separately and combined, paired *t*-tests were used in detecting differences in body composition obtained with both the in-built prediction equations supplied with the Tanita system and the deuterium oxide dilution technique. As the Tanita scales in-built prediction equations only cover 7 years and above, the characteristics of body composition from the deuterium oxide dilution technique was compared with the Tanita system in a subsample (n=66) aged 7 to 10 years. The mean difference is presented for FFM measured by the two techniques.

The bias and limits of agreement (mean difference±1.96 SD) in relation to deuterium oxide dilution were assessed with the Bland-Altman method (29). The recommended approach for comparing two methods is to analyze the differences between the measurements on each subject and use mean of the differences to estimate the average bias of one method relative to the other. If the mean is negligible, the methods generally agree; however, to evaluate how well the methods are likely to agree for an individual, the standard deviation of the differences for each child was used for examining the agreement between the methods. For reasonably symmetric distributions, the range (mean±2 SD) was expected to include about 95% of the observations: this was used in indicating 95% limits of agreement (29). The agreement between FFM derived from the deuterium oxide dilution technique and the Tanita system was evaluated, and Bland-Altman analysis was then used for comparing the agreement for FFM between deuterium oxide dilution technique and the Tanita system.

### Generating novel prediction equations (regression equations)

To create novel prediction equations for estimating FFM in this population, the FFM values derived from the deuterium oxide dilution were used as the reference method and impedance values from the Tanita system in the study sample of Bangladeshi children. The equations were generated by linear regression analysis, and impedance index (height^2^/impedance) was fitted as the primary predictor in the basic model, which was then developed by adding age and sex as further predictors. We excluded 4 FFM outlier's values (4 or more standard deviations from the mean residuals), which were physiologically implausible; hence, these were removed from the regression equations. All the possible regression was used because this method guarantees to find the model having the largest R^2^ and the smallest standard error of the estimate. The R^2^ value was used as an indication of the predictive value of the new equations; R^2^ is simply the squared value of R, also called coefficient of determination and indicates the size of the variation in the dependent variable (FFM) that is explained by the independent variable (height, impedance, weight, age, sex, and 4 site skinfold) in the model. We tried adding skinfolds in the model but they did not help much and probably not much useful in the field as skinfolds are not often measured.

## RESULTS

Descriptive statistics for the 200 subjects, grouped by age and sex, are presented in Table 1. In general, boys had significantly higher weight, BMI, and head-circumference than girls, and girls had larger triceps and subscapular skinfold thickness than boys. Most descriptive variables were significantly different across the age- and sex-groups, with the exception of BMI and MUAC for boys and BMI and all skinfolds for girls (Table 1).

The characteristics of body composition from the deuterium oxide dilution technique were compared with the Tanita scales in-built prediction equations in a subsample (n=66) of age 7 to 10 years (Table 2). For boys, TBW (mean difference=0.62 kg, 95% CI=0.28-0.97, p=0.001) was underestimated by the Tanita system. For girls, there was no difference in TBW measured by both deuterium dilution and the Tanita system. However, FM was underestimated (mean difference=1.06 kg, 95% CI=0.16-1.97, p=0.023) and FFM was overestimated (mean difference= −1.30 kg, 95% CI= −1.95 to −0.64, p=<0.001) by the Tanita system.

**Fig. 1.**
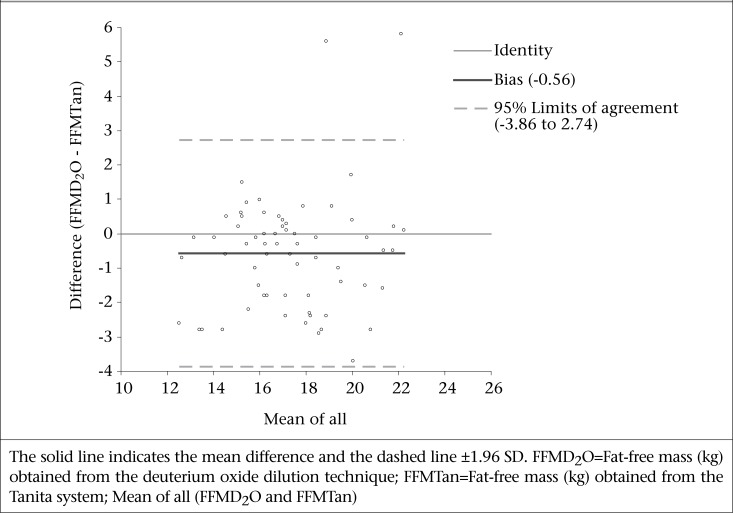
Bland-Altman plot showing agreement in the measurement of FFM between the Tanita system and the deuterium oxide dilution method in a subsample (n=66) of subjects aged 7 to 10 year

The Bland-Altman plot (Figure 1) displays the difference in FFM obtained from the deuterium oxide dilution technique and the Tanita system plotted against the average FFM of both measures. The SD of difference was 1.69 kg, the bias was −0.56, and the 95% limits of agreement (mean difference±1.96 SD) between the methods was −3.86 to 2.74; this reflected an inaccurate estimation of the Tanita inbuilt prediction equation compared to deuterium oxide dilution.

### Best-fit equation for impedance

**Fig. 2.**
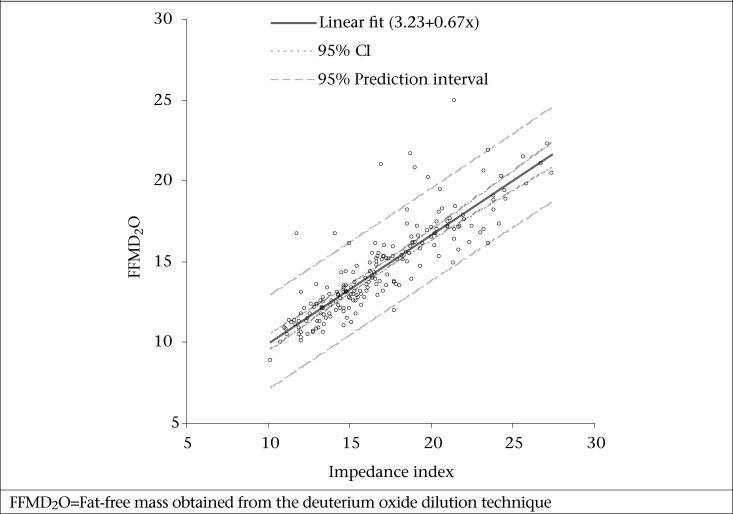
Plot of FFM (kg) from deuterium oxide dilution methods (y-axis) against impedance index (height^2^/Impedance, x-axis) for the prediction of FFM equation

The distribution of data-points between the FFM derived from deuterium oxide dilution technique was plotted against impedance index for the prediction of FFM equation, with children's height and impedance values from the Tanita system (Figure 2). There was an intercept of 3.23 (95% CI 2.30-4.16) and a slope of 0.67 (95% CI 0.62-0.72). Linear regression analysis was used in developing new prediction equations for FFM. First, the impedance index was added as the only independent variable (Table 3), and this basic model with variables height and impedance explained 83% of the variance (adjusted R^2^=0.83, standard error=1.14, p<0.001) in FFM estimated by the deuterium oxide dilution method. However, in the linear regression model, adding weight, age, and sex improves the fit (adjusted R2=89%, Table 3). The best-fit equation to predict FFM from linear regression modelling was achieved by adding weight, sex, and age to the basic model, bringing the adjusted R^2^ to 89% (standard error=0.90, p<0.001).

**Table. 1.** Characteristics of subjects

Age (years)	No.	Weight (kg)	Height (cm)	BMI (kg/m2)	MUAC (cm)	Head circumference (cm)	Impedance (Ohm)	Imped-ance Index	Skinfolds (39)			
									Biceps	Triceps	Sub-scapular	Supra-iliac
Male:												
All	102	16.83±2.9a	111.43±9.6	13.49±0.9a	15.59±1.2	49.34±1.4b	717.2b	17.6a	3.99±0.9	6.39±1.4a	4.49±0.8b	4.33±1.2
4.00 to 4.99	11	13.81±1.3	98.28±3.4	14.27±0.7	15.45±0.9	48.75±1.5	707.1	13.7	5.10±0.8	7.35±1.3	5.13±0.9	5.53±1.9
5.00 to 5.99	47	15.66±2.4	107.32±6.3	13.53±1.1	15.45±1.3	49.00±1.4	722.1	16.2	4.16±0.7	6.71±1.3	4.53±0.7	4.36±1.2
6.00 to 6.99	8	17.03±1.9	112.41±5.7	13.45±0.8	15.50±0.9	49.76±1.3	697.0	18.3	3.73±0.5	5.72±1.2	4.22±0.7	3.78±1.0
7.00 to 7.99	14	18.01±1.5	116.37±4.6	13.30±0.8	15.76±0.9	49.89±0.9	715.1	19.0	3.81±0.8	6.20±1.38	4.54±0.9	4.21±1.1
8.00 to 8.99	12	19.17±2.1	121.00±5.9	13.06±0.7	15.52±0.7	49.81±1.5	720.0	20.6	3.48±0.5	5.40±0.95	4.08±0.5	4.08±0.8
9.00 to 10.00	10	21.11±2.6	125.99±4.5	13.27±1.2	16.32±1.5	49.86±1.4	721.1	22.2	3.09±0.6	5.81±1.74	4.22±0.7	3.81±0.8
P-valuesc		<0.001	<0.001	0.061	0.429	0.055	0.925	<0.001	<0.001	0.003	0.018	0.010
Female:												
All	98	15.93±3.3	109.51±9.8	13.16±0.8	15.51±1.3	47.97±1.8	767.5	15.9	4.21±0.8	6.91±1.5	5.02±0.9	4.66±1.3
4.00 to 4.99	13	13.28±1.1	99.25±1.8	13.47±0.9	15.09±1.1	46.69±2.9	770.9	12.8	4.62±1.2	7.89±1.5	5.57±1.1	5.45±1.9
5.00 to 5.99	42	14.26±1.9	104.73±5.9	12.94±0.7	15.06±1.1	47.80±1.5	782.2	14.2	4.29±0.7	6.84±1.2	4.97±0.9	4.51±0.9
6.00 to 6.99	13	15.63±1.2	109.38±2.6	13.06±0.8	15.25±0.9	47.57±0.8	741.4	16.5	4.01±0.7	6.49±1.2	4.77±0.9	4.34±0.8
7.00 to 7.99	8	16.93±1.9	114.46±4.9	12.93±1.4	15.45±1.5	48.58±0.9	812.3	16.4	3.93±0.7	6.44±1.5	4.45±0.9	4.18±0.9
8.00 to 8.99	11	20.07±2.6	122.29±6.9	13.39±0.9	16.73±1.3	49.41±1.8	760.3	19.8	4.25±0.7	7.07±1.9	4.92±1.0	4.68±1.1
9.00 to 10.00	11	20.96±2.5	123.65±6.9	13.68±0.7	16.85±0.7	48.73±1.1	712.8	21.7	3.78±0.7	6.67±1.7	5.35±1.1	5.01±1.8
P-valuesc		<0.001	<0.001	0.061	<0.001	0.002	0.023	<0.001	0.108	0.132	0.113	0.127

BMI=body mass index;

MUAC=mid-upper arm-circumference;

Reported values are means±SD;

aMeans are significantly different from girls at p<0.05;

bMeans are significantly different from girls at p<0.001;

cOne-way ANOVA for differences across age-groups;

ANOVA=Analysis of variance

**Table. 2.** Comparison between deuterium oxide dilution technique and Tanita system in measuring body composition in a subsample (n=66)^a^ of subjects aged 7 to 10 years

Body composition	Deuterium oxide dilution	Tanita system	Mean difference (95% CI)	p value
Overall (n=66)				
TBW (L)	13.10±1.99	12.89±1.78	0.22 (-0.09, 0.52)	0.158
FFM (kg)	17.04±2.65	17.59±2.43	-0.56 (-0.98, −0.14)	0.009
FM (kg)	2.36±1.65	1.89±1.10	0.47 (-0.02, 0.95)	0.060
BF%	13.09±6.06	9.10±3.14	3.99 (2.31, 5.67)	<0.001
Boys (n=36)				
TBW (L)	13.29±1.91	12.67±1.60	0.62 (0.28, 0.97)	0.001
FFM (kg)	17.35±2.53	17.30±2.18	0.05 (-0.41, 0.51)	0.827
FM (kg)	1.91±1.48	1.94±.56	-0.03 (-0.49, 0.42)	0.883
BF%	10.76±5.23	10.16±2.58	0.60 (-1.15, 2.34)	0.490
Girls (n=30)				
TBW (L)	12.88±2.11	13.15±1.97	-0.27 (-0.76, 0.23)	0.278
FFM (kg)	16.66±2.78	17.96±2.69	-1.30 (-1.95, −0.64)	<0.001
FM (kg)	2.90±1.71	1.83±1.53	1.06 (0.16, 1.97)	0.023
BF%	15.89±5.87	7.81±3.31	8.08 (5.73, 10.44)	<0.001

BF%=body fat percentage;

CI=confidence interval;

FFM=Fat-free mass;

FM=fat mass; TBW=Total body water p values refer to paired t-tests;

Values are mean±SD;

^a^Comparison conducted on a subsample of individuals because the Tanita system equations include children aged 7 years and above

**Table. 3.** Prediction equations for FFM in rural Bangladeshi children produced by linear regression modelling

Model	*Equation	Adjusted R2	Standard error	C*p*
Basic model	FFM=3.21+0.66 (height2/impedance)	0.83	1.137	26.64
+Weight	FFM=1.40+0.53 (weight)+0.25 (height2/impedance)	0.88	0.943	4.45
+Sex and age	FFM=2.34+0.50 (weight)–0.52 (sex)+0.18 (age)+0.21 (height2/impedance)	0.89	0.902	3.51

FFM=fat-free mass

*To develop prediction equations for estimating FFM, the FFM from the deuterium oxide dilution and impedance values from the Tanita system were used. The equations were generated by linear regression analysis with fitted impedance index (height2/impedance) as the primary predictor, and weight, age, and sex measurements as further predictors

## DISCUSSION

In the current study, the ability of the in-built equations from the Tanita TBF-300A system to assess body composition of rural Bangladeshi children aged 7-10 years was investigated, with deuterium oxide dilution as a reference method. The equation for predicting FFM in this population for the age interval 4-10 years was developed with impedance index and age.

The in-built Tanita equation measured body composition in this population with significant bias for both sexes. The bias with the in-built prediction equation indicated that the Tanita system was inaccurate for Bangladeshi children aged 7–10 years, although the Tanita system agrees well with reference methods in Caucasian populations (12). The Tanita system underestimated TBW in boys and underestimated BF% in girls. This variability is probably due to differences in physique and body geometry between the Bangladeshi and European children used when deriving the Tanita equations (30). Previous studies have reported that BIA methods overestimate BF% in lean subjects (31,32) and underestimate at higher BF values (32,33). The South Asian malnourished phenotype may be characterized by relatively low muscle mass, a maintained high percentage of body fat, and a tendency to central adiposity (4,34).

When developing novel equations for the prediction of FFM in this population, the basic model that used the variables height and impedance explained 83% of the variance in results from the deuterium dilution technique. The addition of weight, sex, and age to the basic model further increased R^2^ to 0.89, thus, the equation was considered the best-fit equation to predict FFM. The fit obtained was similar to the novel equation developed for a Tanita BC-418MA segmental body composition analyzer used in rural Gambian children (R^2^=0.81) (20).

The disadvantage of the BIA method is that TBW or FFM are estimated by mathematically-derived inbuilt equations, the majority of which are derived from West European and North American populations. The validity of the BIA method for estimating TBW or FFM in populations and age-groups that are different from those used in developing the in-built equation has been questioned previously (35); therefore, age- and population-specific equations may improve the validity. The independent variables used in the final prediction equation had a high degree of association with the dependant variable and were comparable with published data (27,36,37). However, the novel prediction equations derived in the current study are only applicable within the same or similar populations and age range.

The deuterium oxide dilution, used as a reference method, measures total body water converted into FFM and, thus, is not an ideal gold standard for body fat measurement. Ideally, a four-component model should be used as a reference method but this was impossible in the rural field setting in which study was performed. Deuterium oxide dilution is relatively easy to perform but has some limitations, including the assumption of the hydration of FFM, which may vary with age, sex, maturation, and ethnicity (14,38,39). To estimate FFM from TBW, age- and sex-specific hydration fractions (26) were used; however, these factors were not ethnicity-specific. There is a lack of information on the hydration of fat-free tissue in Bangladeshi children. However, there is little indication of ethnicity-specific variation in hydration (14).

The use of deuterium oxide dilution may be considered a strength of this study as the technique is appropriate for the rural setting and is a well-accepted measure for a reference method. The saliva collected for deuterium oxide dilution assay in this study closely resembled results obtained from serum (40). The children sampled were not balanced within all age-groups as there were more children in the age range 5 to 5.99 years.

### Conclusions

This study has demonstrated that the measurement of FFM in Bangladeshi children aged 4-10 years, using an established BIA analyzer, may not be wholly accurate, thus questioning the usefulness of this technique for the assessment of the important disease risk factor. As an alternative, we have generated novel prediction equations for FFM based on height, impedance, weight, sex, and age measurements for use in this population.

## ACKNOWLEDGEMENTS

We thank the participants and their families for their involvement in the study, the field-team members for their excellent work, and the data management people.

The study was funded by icddr,b; United Nations Children's Fund; Swedish International Development Cooperation Agency (Sida); UK Medical Research Council; Swedish Research Council; Department for International Development (DFID); Japan Society for the Promotion of Science; Child Health and Nutrition Research Initiative; Uppsala University; and US Agency for International Development. icddr,b acknowledges the following donors who provided unrestricted support: Australian Agency for International Development; Canadian International Development Agency; DFID; Government of the People's Republic of Bangladesh; Kingdom of the Netherlands; Swiss Agency for Development and Cooperation; and Sida. The International Atomic Energy Agency (IAEA) also supported this study.
